# Generation of Recombinant Primary Human B Lymphocytes Using Non-Viral Vectors

**DOI:** 10.3390/ijms22158239

**Published:** 2021-07-30

**Authors:** Daniel Keim, Katrin Gollner, Ulrich Gollner, Valérie Jérôme, Ruth Freitag

**Affiliations:** 1 Process Biotechnology, University of Bayreuth, 95447 Bayreuth, Germany; daniel.keim@uni-bayreuth.de (D.K.); valerie.jerome@uni-bayreuth.de (V.J.); 2 Praxis am Schießgraben, Schießgraben 21, 95326 Kulmbach, Germany; k.jaekel@yahoo.com (K.G.); gollner@hno-operationen.de (U.G.)

**Keywords:** non-viral gene delivery, human B lymphocytes, primary cells, poly(2-dimethylamino) ethyl methacrylate, polycation, nano-star

## Abstract

Although the development of gene delivery systems based on non-viral vectors is advancing, it remains a challenge to deliver plasmid DNA into human blood cells. The current “gold standard”, namely linear polyethyleneimine (l-PEI 25 kDa), in particular, is unable to produce transgene expression levels >5% in primary human B lymphocytes. Here, it is demonstrated that a well-defined 24-armed poly(2-dimethylamino) ethyl methacrylate (PDMAEMA, 755 kDa) nano-star is able to reproducibly elicit high transgene expression (40%) at sufficient residual viability (69%) in primary human B cells derived from tonsillar tissue. Moreover, our results indicate that the length of the mitogenic stimulation prior to transfection is an important parameter that must be established during the development of the transfection protocol. In our hands, four days of stimulation with rhCD40L post-thawing led to the best transfection results in terms of TE and cell survival. Most importantly, our data argue for an impact of the B cell subsets on the transfection outcomes, underlining that the complexity and heterogeneity of a given B cell population pre- and post-transfection is a critical parameter to consider in the multiparametric approach required for the implementation of the transfection protocol.

## 1. Introduction

B lymphocytes (B cells) are a key component of the human immune system. They can be isolated from peripheral blood or tonsils and be expanded in culture after activation by mitogenic agents [1,2]. In recent years, the interest for genetic modification of human B cells has been growing, since B cells, in particular, those of the plasma cell subtype, are long-lived and have high protein production potential [3]. Recombinant plasma B cells could thus, in future, be harnessed as “living drug delivery devices” for in vivo applications [4,5,6,7,8,9]. Such an approach would improve current therapies for autoimmune diseases [10], where repeated injections of neutralizing mAb are otherwise necessary to maintain a therapeutic titer. B cell engineering could also provide methods for vaccination against viruses such as HIV [11,12,13] or lead to alternative therapies for the neutralization of overexpressed cytokines in chronic inflammatory diseases such as rheumatoid arthritis [8]. In the treatment of genetic disorders, such as hemophilia B, engineered B cells could replace the costly periodic enzyme replacement therapies [4,14].

Finally, several recent publications show that murine and human B cells can be efficiently edited by the clustered regularly interspaced short palindromic repeats (CRISPR)–CRISPR-associated protein 9 (Cas9) system (CRISPR/Cas9) [4,12,13,14,15,16]. The examples range from the replacement of the endogenous antibodies by antibodies targeting viruses [12,17] to the creation of chimeric B cell receptors presenting synthetic antigens [8,12,13,18]. However, in order to use CRISPR-Cas9 in B cells, it is necessary to first deliver DNA encoding for the nuclease Cas9 and the synthetic guide RNA to the cells, where they are not naturally present [19,20]. So far, in vitro delivery strategies for CRISPR/Cas9 into human B cells rely on viral vectors (mostly adenovirus, adeno-associated virus, lentivirus), which have high transfection efficiencies but are associated with a limited carrier capacity, immunogenicity, and the risk of insertional mutagenesis (for a review see [14,20,21]). Non-viral gene delivery systems have been suggested as alternatives but, in the case of B cells, are severely limited by low transfection efficiency and high cytotoxicity [20,21]. 

Better results were achieved with physical methods such as electroporation. Li et al., 2006, reached a transfection efficiency of 65% for a pDNA encoding for CD154 cDNA in chronic lymphocytic leukemia B cells (B-CCL), albeit with survival rates of only 30% 24 h post-transfection [22]. Optimizing the salt concentration and the amount of pDNA may improve the efficacy of electroporation in peripheral primary B cells since, e.g., Canoy et al. reached 80% transfected cells at 55% viability [23]. Moghimi et al. showed that Nucleofection, a specialized form of electroporation, may yield up to 65% transfected cells with 70% viability, whereas a liposome-based vector tested in parallel (i.e., Lipofectamine) never achieved more than 5% of transfected B cells [24]. Such reports demonstrate that electroporation is at present the method of choice for non-viral gene delivery into B cells. However, as taken from these reports, electroporation requires large numbers of cells (up to 5 × 10^7^ cells per electroporation mixture), which might not always be extractable from primary tissue material of a single donor. In addition, extensive amounts of pDNA are also needed (up to 440 µg pDNA per electroporation mixture), yet the production of high-quality pDNA is time-intensive and costly. In consequence, there is still a serious need for alternative non-viral procedures for gene delivery into primary B cells.

Cationic polymers represent a popular group of non-viral transfection agents for mammalian cell lines but are usually less capable of transfecting primary (immune) cells [25,26]. An early report showed that low levels of transfection can be reached in primary murine splenic B lymphoblasts (i.e., LPS-activated B cells) using diethylaminoethyl (DEAE)-dextran [27]. To the best of our knowledge, l-PEI and poly-2-(dimethylamino)ethyl methacrylate (PDMAEMA), i.e., two current standard polycations for the transfection of mammalian cell lines [28,29,30], have never been proposed in the pertinent literature for the transfection of B cells with pDNA. In this context, PDMAEMA is a particularly interesting base structure for the design of improved non-viral agents because its chemistry allows precise control over molecular weight and structure during synthesis via atom transfer radical polymerization (ATRP) [31]. In the past, we have proposed non-linear PDMAEMA nano-structures (stars, grafts) as efficient agents for nucleic acids delivery [32,33,34,35] to “hard-to-transfect” cells, including primary human T lymphocytes [36,37]. Recently, we showed that the superior transfection capability of nano-stars relies on the destabilization of plasma and nuclear membranes, which presumably leads to transient pore formation [38], i.e., follows a transfection mechanism akin to electroporation.

In this publication, we seek to implement a non-viral polycation-based procedure for the efficient delivery of pDNA into human primary B cells derived from tonsillar tissue. As shown in the past, the development of an effective transfection protocol for hard-to-transfect cells requires a multiparameter approach. In particular, the polymer density (µg per 10^6^ cells), the polymer concentration (µg per mL), the cell density during the transfection, as well as the contact time between DNA/polycation polyplexes and cells, are critical for the transfection outcome (transfection efficiency, cell viability) [36]. Moreover, preparations of tonsillar B cell pools contain several B cell subpopulations that further differentiate upon cultivation in the presence of the mitogenic agents [39]. Compared to transfection of cell lines, the non-clonality of the primary B cells introduces an additional parameter that needs to be considered during protocol development. 

## 2. Results and Discussion

### 2.1. Comparison of PDMAEMA-Nano-Stars to l-PEI for the Transfection of Primary Human B Lymphocytes

First, a standard approach to the development of a transfection protocol was used for both l-PEI (25 kDa), as the current gold standard in polycationic transfection agents, and the PDMAEMA based nano-stars. In this approach, the amount of pDNA was kept constant (3 µg), and the N/P ratio was adjusted by varying the amount of polycation used for polyplex formation. To get enough cells for parallel analysis of several transfection conditions (e.g., the N/P ratios), it was first necessary to expand the cells in growth medium for several days. To this end, the cells were cultivated with mitogenic factors (i.e., CD40L, IL-4, IL-21). Usually, four to six days were necessary to produce the required number of cells. Moreover, active division, which is accompanied by a temporal disassembly of the nuclear membrane, is generally accepted as a facilitator of transfection [40]. 

**Figure 1:**
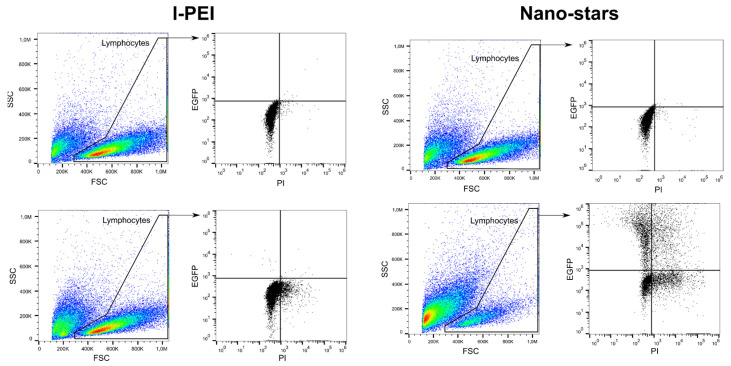
Representative gating strategy for flow cytometry analysis. Top: mock transfection, bottom: Cells transfected with l-PEI (**left**) and nano-stars (**right**).

In this set of experiments, cells were transfected after four days of culture and incubated for four hours with the polyplexes in 6-well plates (transfection volume: 2 mL). The transfection outcomes (TE, survival) were analyzed 24 and 48 h post-transfection (Table 1) by flow cytometry (“Lymphocytes” gate). The gating strategy used in this process is shown in Figure 1.

No replicates were tested since the number of primary cells from a given donor is, regardless of the expansion step, limited, while cells from different donors must be expected to vary in their experimental response [39]. We limited our testing to N/P ratios corresponding to polymer concentrations ≤40 µg mL^−1^ for nano-stars and ≤4 µg mL^−1^ for l-PEI to avoid possible cytotoxic effects.

For the nano-stars, increasing the N/P ratio led to an increase in the TE with a concomitant decrease in viability. An N/P ratio of 20 allowed reaching a TE of ca. 20% in cultures 24 h post-transfection; however, only half of the cells survived. In terms of expression level, an N/P ratio of 7.5 seems to lead to the highest GFP production. In the case of l-PEI, the TE was always in the single-digit range, while the survival rate was around 80%. These results follow the general observation that high transfection efficiency is usually linked to greater cytotoxicity (as reviewed by Zhang et al., 2017) [41]. Interestingly, whereas the TE decreased rapidly for both transfection agents with the cultivation time post-transfection, reaching values <1% for l-PEI and <10% for the nano-stars after 48 h post-transfection, the expression level, indicated by the median fluorescence intensity (MFI), increased in all cases. This result indicates that the remaining transfected living cells are transcriptionally active. 

In our experimental setup, transfection was supposed to be transient, i.e., no active integration into the genome was intended. However, such a rapid decrease in TE was not expected and is usually not observed in cell lines transfected according to similar protocols, where GFP accumulation can typically be observed for at least 72 h [42]. An explanation for this behavior can only be speculated upon. In the past, Seiffert et al. reported that circular pDNA induces apoptosis in nucleofected primary B cells [43]. It has also been reported that exposure of cells to apoptotic stimuli induces a rapid loss of cell volume, the so-called apoptotic volume decrease [44]. Since we restricted our analysis to the lymphocyte population identified by scattering properties, a significant decrease in the cell volume during the incubation post-transfection would lead to a shift of these cells outside of the “Lymphocytes” gate (i.e., smaller forward scatter) and *inter alia* decrease the TE evaluated in this gate. 

The better survival of the cells in case of transfection with l-PEI may also be ascribed to the lower polymer densities (6.0 to 39.0 µg per 10^6^ cells for l-PEI, 22.0 to 144.0 µg per 10^6^ cells for the nano-stars) and polymer concentrations (0.6 to 4.0 µg mL^−1^ for l-PEI, 2.0 to 14.0 µg mL^−1^ for the nano-stars) required to reach the indicated N/P ratios (see Table S1 for details). However, for both polycations, the polymer concentration at the highest N/P ratio was still below the LD_50_ values recorded for free l-PEI (12 µg mL^−1^) and nano-stars (39 µg mL^−1^) in L929 cells (MTT assay) by our group [45]. Previously, we have shown that human primary T cells have a two-fold higher sensitivity to these polycations than the L929 cells and some similarity can be presumed for primary B cells [37]. Still, none of the polymers were expected to be toxic in the concentration range tested. Since cells generally tend to tolerate nano-stars better than l-PEI, the negative effects on viability observed here for the nano-stars may be associated with cellular “disorders” post-engulfment of the polyplexes, which built up during the post-transfection period. This is corroborated by data recently obtained by us for Jurkat cells [38]. A similar effect is not expected for l-PEI, which has a much lower tendency to enter the cells (low TE) and thus is removed during washing.

### 2.2. Influence of the Cell Number and the Polymer Density (Amount per Cell)

In the past, we were able to show that changing the geometry of the transfection vessel from plate to tube allows reducing the reaction volume, thereby *inter alia* intensifying the interactions between cells and polyplexes, while concomitantly accelerating the transfection kinetics. As a result, the tube transfection protocol highly improved the transfection outcomes of some “hard-to-transfect” cells in terms of TE and viability post-transfection [36]. Here, we hypothesized that a similar benefit may be possible in the case of the primary B cells. For fine-tuning the conditions, we investigated the influence of the cell number, but also the polymer density (µg per 10^6^ cells) using quantities identified as most efficient for gene delivery into human T cells as starting conditions [36]. 

**Figure 2:**
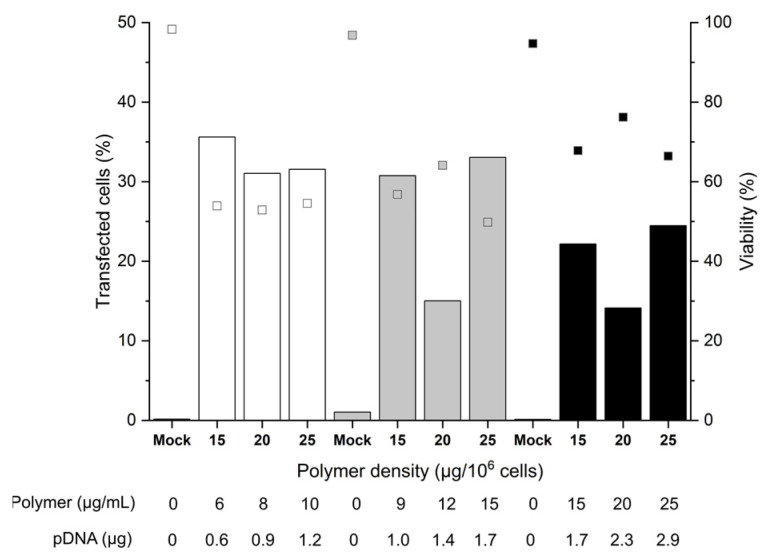
Influence of cell number and polymer density on transfection efficiency and viability. Transfection in tubes at day 6 post-thawing. Cell numbers during transfection were: 2 × 10^5^ (white), 3 × 10^5^ (grey), 5 × 10^5^ (black) cells. pDNA corresponds to the amount of plasmid per experiment. Contact time: 90 min. N/P: 10, transfection volume: 0.5 mL. TE (bars) and viability (squares) measured 48 h post-transfection. “Mock”: cells subjected to mock transfection. *n* = 1. Cell viability on the day of transfection: 81%.

For the investigation of the cell number, 2, 3, or 5 × 10^5^, cells were incubated for 90 min with polyplexes formed at an N/P ratio of 10, i.e., a ratio sufficient for charge neutralization of the pDNA when using nano-stars [36]. Since preliminary screening experiments had shown that polymer densities ≤ 10 µg polymer per 10^6^ cells (≤4 µg polymer per mL) led to TE < 15%, experiments were carried out at polymer densities of 15 to 25 µg polymer per 10^6^ cells (6 to 25 µg polymer mL^−1^), the N/P ratio of 10 was assured in each case via the added pDNA. Transfection efficiencies (TE) and the cell viabilities were analyzed 48 h post-transfection by flow cytometry (Figure 2), as prescribed by Riedl et al. for primary T cells [36]. If anything, a measurement after 48 h constitutes a “worst case scenario”, since in the experiments described above, the TE tended to decrease with post-transfection cultivation time. 

A maximum TE of 36% was achieved with 2 or 3 × 10^5^ cells per tube, which was a considerable improvement over the plate protocol, where TEs generally were below 10% after 48 h. A further increase in the cell number to 5 × 10^5^ cells per tube reduced the TE 1.8-fold. This could be related to insufficient mixing of cells and polyplexes due to the higher viscosity of the mixture. Strikingly, for 3 and 5 × 10^5^ cells, at identical polymer concentration (15 µg mL^−1^) and pDNA amount (1.7 µg), a significant increase in TE/decrease of viability correlated with an increase in the polymer density during transfection. Hence, an increase in the number of polyplexes per cell (i.e., polyplexes dose) improves the TE to the detriment of cell viability.

On the other hand, if we assume the otherwise difficult to explain lower TEs measured with 20 µg polymer per 10^6^ cells for 3 and 5 × 10^5^ cells per tube to be due to experimental error, the polymer density has no major influence on the transfection outcomes for a given number of cells. In the past, we observed similar trends for the Jurkat cells [36]. The fact that increasing the polymer density (i.e., the polyplex dose, but also the pDNA dose) for a given number of cells does not influence the overall TE, could be a first indication that some saturation of one or several rate-limiting steps of the transfection (cellular and/or nuclear uptake, decomplexation of polyplexes, or other) occurs. Hence, for a given polymer density, there is an upper limit of effectivity above which further raising of the polymer density has no beneficial effect and only reduces the cell survival. Furthermore, overloading the transcriptional machinery due to an excess of pDNA in the nucleus (i.e., a titration of the transcription factors) cannot be excluded. On the other hand, a decrease in the relative polyplex dose via increasing the cell number at a given amount of polyplexes negatively influences the TE. We can hypothesize that in that case, the average number of polyplexes per cell is reduced (in line with the observed trend towards improved viabilities), which leads to a reduced level of GFP expression. In general, cells expressing GFP can be readily detected by flow cytometry. However, a precise distinction of cells with low expression levels from the autofluorescence of non-transfected cells is difficult due to a distribution overlap. Hence, the observed TE might be underestimated. 

The survival rates of the B cells showed some variability (between 50 to 70%) but were tendentially better than in the plate protocol, despite the improved TEs. The mock-transfected cells exhibited viabilities > 90%, indicating that the transfection procedure per se had no negative effect on B cell viability within the 48-h time frame of observation. Remarkably, within the range tested, the polymer concentration adjusted during transfection did not itself influence the cell viability. This first set of data indicated that 2 × 10^5^ cells per tube and polymer densities between 15 and 25 µg mL^−1^ are well-suited conditions that can be taken as a basis for further optimization.

### 2.3. Influence of the Polyplex Exposure and the Recovery Times in B Cell Transfection

**Figure 3:**
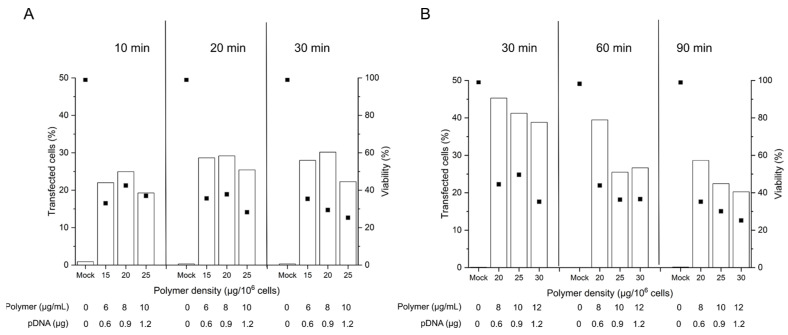
Influence of the polyplex contact time on transfection efficiency and viability. Cell number during transfection: 2 × 10^5^ cells (day 4 post-thawing), tube transfection protocol. pDNA corresponds to the amount of plasmid per tube. Contact time: as indicated, N/P: 10, transfection volume: 0.5 mL. TE (bars) and viability (squares) were measured 48 h post-transfection. Mock: cells subjected to mock transfection. *n* = 1. Cell viability on the day of transfection: 80%, group A (**A**) and 93%, group B (**B**).

Considering that the nano-stars induce a transient poration of the plasma membrane [38], the prolonged 90 min-exposure to the polyplexes as prescribed in the standard protocol might have had negative consequences in sensitive primary cells, which have a less robust plasma membrane repair machinery than, e.g., cancer cells [46]. To test the hypothesis that a reduction in the contact time may be of benefit, 2 × 10^5^ cells were incubated for 10 to 90 min with polyplexes (N/P 10, corresponding to 15 to 30 µg polymer per 10^6^ cells or 6 to 12 µg polymer mL^−1^). The limited cell number recovered from individual batches after freezing/thawing obliged us to do the testing on two batches of thawed cells (same donor) to cover the entire time range we wanted to study (group A: 10 to 30 min, group B: 30 to 90 min). Experiments were started as soon as sufficient biomass was available in the sum of the two batches, i.e., on day 4 post-thawing. Transfection efficiencies and cell viabilities were again measured 48 h post-transfection by flow cytometry (Figure 3).

Even though issued from the same donor tissue, the obtained B cell pools showed differences in the overall viability on the day of transfection, which was 80% in group A and 93 % in group B. As reported in the past, B cells show some susceptibility to freezing damage [47], which may influence their fitness after thawing. In consequence, we did our best to standardize the procedures, however with mitigated success.

The TEs obtained in this experiment for the 90 min incubation were in the range shown before for cells were transfected 6 days post-thawing (Figure 2), which suggests that a reduction in the cultivation time before transfection has little effect on the transfection outcome in terms of TE. Lowering the polyplex exposure time from 90 to 60 and 30 min has a beneficial effect on the TE as well as on the viability of the cells, while yet lower exposure times show no additional benefit. A 30 min exposure to the polyplexes may thus be considered an optimum contact time for the transfection of primary B cells according to the tube protocol. However, under experimentally similar conditions, group A exhibited lower TEs and viabilities than group B for a given polymer density at 30 min exposure. This may well be due to the lower fitness (viability) of the cells from group A on the day of transfection. In cell lines, a viability of >90% is typically prescribed in standard non-viral transfection protocols, a quality, which is not always achievable in primary cells.

To further investigate the extent of possible intra- and inter-experimental variations, cells recovered from five cryovials (same donor) were transfected after 4 days of cultivation in the presence of mitogens as follows: 2 × 10^5^ cells were incubated for 30 min with polyplexes corresponding to 15 µg polymer per 10^6^ cells (6 µg polymer mL^−1^, N/P ratio: 10). Transfection efficiencies and cell viabilities were measured 48 h post-transfection (Table 2). Whereas the intra-experimental variation (technical replicates) was low (3.6% for TE; 1.4% for viability), the inter-experimental variation (experimental replicates) was more pronounced (9.2% for TE; 11.6% for viability). It should be noted that in these experiments as well, higher viability on the day of transfection seemed to correlate with an improved transfection outcome.

Finally, the transfection capability of l-PEI in the tube protocol was tested. In these experiments, we kept the experimental setup as similar as possible to the conditions used for nano-stars (Table 2) but extended the range of N/P ratios tested. After 48 h of cultivation post-transfection, the transfection efficiency was below 0.5% in all cases with viabilities ≥73% (detailed values are given in Table S2). These results underline that a switch to the tube protocol has no benefit in the transfection of B cells with l-PEI.

To fully investigate any effect of the post-thawing recovery time on the day of transfection, cells expanded from various cryovials from the same donor were transfected three to five days post-thawing. During this time interval, the cells were in the exponential phase (growth rate: 0.071 h^−1^). A representative growth curve is shown in Figure S1. The TE and the cell viabilities were analyzed 24 to 48 h post-transfection in order to concomitantly follow the development of these two parameters with cultivation time, Table 3. Again, a pronounced heterogeneity was observed between the batches thawed, even though the cryovials were prepared from the same pool of cells isolated from one donor. Cell viability, in particular, varied from 65 to >90% after three to five days of cultivation post-thawing. To reduce at least the influence of the cell viability on the day of transfection, we only used the expanded cells if their viability was >80%. 

According to the results compiled in Table 3, the cells seemed to be most tolerant towards the transfection conditions on day 4, since the viability was highest. TEs were in that case similar to day 3, while TE levels of GFP expression and viability dropped for cells transfected on day 5. However, the trend towards a higher GFP expression when the cells were transfected at day 4 post-thawing was not statistically significant. As observed before for transfection, according to the plate protocol, both the TE and the viability of the cells tended to deteriorate 48 h post-transfection compared to the values recorded after 24 h. The difference was statistically relevant for cells transfected on day 3 of cultivation and may thus indeed present a general problem in B cell transfection.

### 2.4. Influence of the Amount of pDNA and Transgene Expression on the Cell Survival

The persistently low viability of the transfected B cells even after 48 h of cultivation post-transfection was not expected. As already discussed above, for most previously investigated cell types, viabilities tended to improve post-transfection, indicating cell recovery. However, it is possible that in the case of B cells, the transfection agent directly interferes with cell division. An early work from McMahon et al. showed that the presence of the polycationic diethylaminoethyl (DEAE)-dextran decreases the proliferative responsiveness of murine splenic B cells 2 to 3-fold [27]. Unspecific effects on the proliferation of peripheral immune cells post-transfection have also been reported, e.g., for Lipofectamine™ 2000 und PEI [48]. 

A reduced survival (<40%) of B cells after transfection has been reported before, e.g., for Nucleofection, and been ascribed to the expression of the reporter transgene GFP [22,49]. Some cytotoxicity of GFP has also been discussed in the past for primary hepatic cells [50,51] and could also be at least partly responsible for the elevated mortality observed here for the transfected B cells. Other publications focusing on Nucleofection report that the survival of primary B cells (murine and human) is inversely proportional to the amount of pDNA applied (2 to 10 µg per 10^6^ cells) [24,43]. In electroporated mesenchymal stem cells, TE and survival were inversely correlated with the physical size of the transfected pDNA molecule [52]. Assuming some parallelism between the mechanisms of electroporation and transfection by nano-stars, similar effects of the pDNA on cell viability can be expected, in particular, since an N/P ratio of 10, as adjusted during our transfection experiments, corresponds to 3 µg pDNA per 10^6^ cells and consequently is in a range described as toxic by some of the above-cited authors. However, as previously shown by our group for Jurkat cells, the amount of pDNA present during transfection influences the level of expression of the transgene and its accumulation (GFP t_1/2_: 26 h, [53]) in the cytoplasm [32]. A similar development can also be assumed for the B cells.

**Figure 4:**
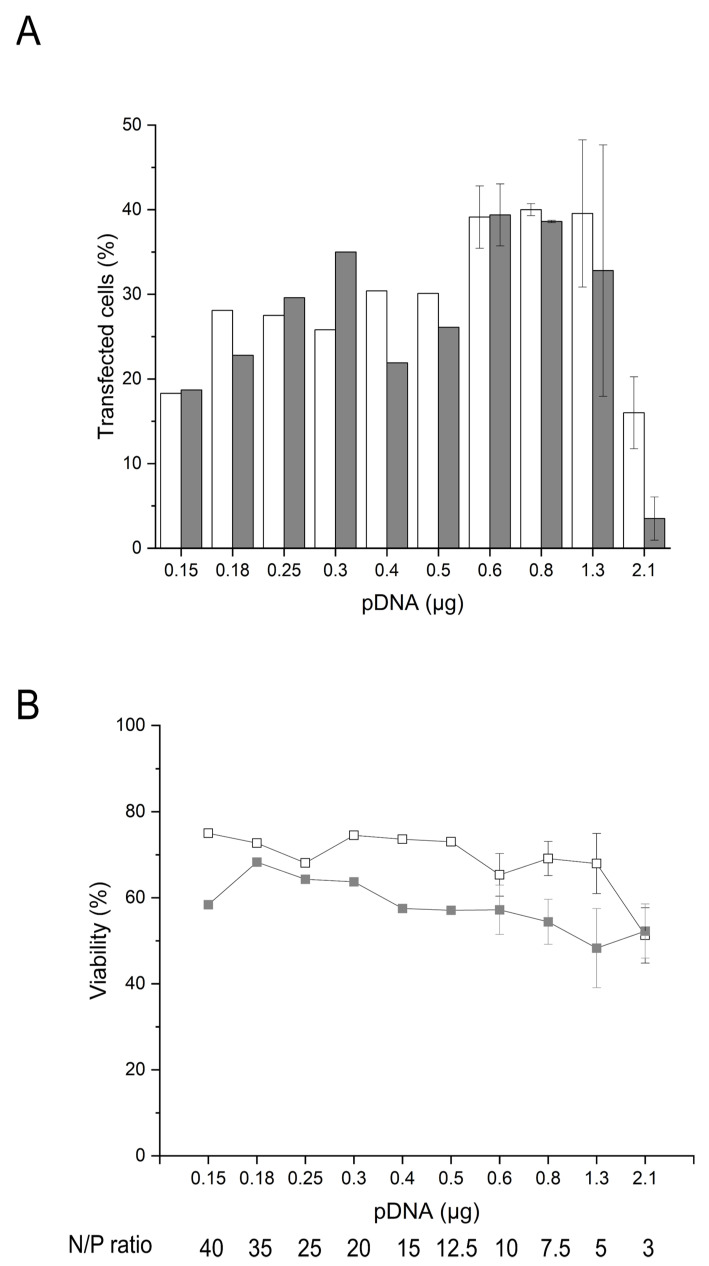
Influence of pDNA amount and cultivation post-transfection on transfection efficiency and viability. Transfection in tubes day 4 post-thawing. TE (**A**) and viability (**B**) measured 24 h (white) and 48 h (grey) post-transfection. Cell number during transfection: 2 × 10^5^ cells. Polymer density: 15 µg mL^−1^, polymer concentration: 6 µg mL^−1^. Contact time: 30 min. Transfection volume: 0.5 mL. Viability of the mock-transfected cells: 97.9 ± 0.3 % (24 h); 97.9 ± 1.4 % (48 h). Cell viability on the day of transfection: >80%. Shown are mean values ± SD, *n* ≥ 2 (N/P ratio 3 to 10) and results of a single transfection (N/P ≥ 12.5, *n* = 1).

Based on these considerations, we analyzed the influence of changing pDNA amounts during nano-star transfection on TE and viability 24 and 48 h post-transfection. Since TE is indicated by the expression of GFP, this also allows a correlation of viability with the amount of GFP present in the cells (i.e., expression level). As pointed out above, increasing the amount of pDNA is expected to raise the GFP expression, which makes a complete unraveling of the potential toxicities of GFP vs. pDNA difficult. However, in the past, Seiffert et al. demonstrated that the pDNA cytotoxicity was not related to the selected transgene cDNA present in the plasmid (i.e., GFP vs. CD79b) [43]. In the subsequent transfection experiments, the amount of pDNA was reduced while keeping the polymer density/polymer concentration constant (15 µg polymer per 10^6^ cells, 6 µg mL^−1^), i.e., by increasing the N/P ratio. Thereby, the polymer concentration was kept below the LD_50_ of the nano-stars, and hence, any observed changes in viabilities were ascribed directly (or indirectly through the transgene expression) to the increasing amounts of pDNA. Four days post-thawing, the cells were incubated for 30 min with polyplexes formed at N/P ratios of 3 to 40, corresponding to 0.2 to 2.1 µg pDNA per tube, Figure 4. 

Under conditions of varied pDNA amount, the cultivation time post-transfection (24 vs. 48 h) had no major impact on the TE except for the lowest N/P ratio tested (N/P 3), where it was again lower after 48 h than after 24 h. (Figure 4A). Maintained or increased TE levels thus were as originally expected, since the GFP expression should be actively driven by the CMV promoter in all cells, having taken up the pDNA, while the GFP protein is highly stable (t_1/2_: 26 h in mammalian cells) towards proteolysis [53,54], which leads to a continued cytoplasmic presence of GFP independently of active transcription [55]. 

On the other hand, and similar to the B cells transfected in plates (Table 1), the viabilities of the B cells transfected according to the tube protocol decreased between 24 h and 48 h post-transfection for all N/P ratios tested except again for N/P 3 (Figure 4B). The B cells thus seem to be unable to recover from the transfection stress, while most other cell types do. Similar trends have recently been observed for human peripheral B cells after electroporation [23]. Most importantly, the viabilities determined 48 h post-transfection, and to a lesser extent, those after 24 h tended to decrease with increasing amounts of pDNA. The level of GFP expression showed a similar dependency on the pDNA concentration (Figure S3A,B). The direct correlation between GFP expression and the PI fluorescence indicative of cell death is shown in Figure S3C,D. Hence, the compromised B cells’ survival post-transfection could eventually be ascribed to a cytoplasmic accumulation of GFP. While the toxicity of GFP may present a problem during the development of a transfection protocol, no similar toxicity needs to arise from other transgenes.

However, as reported in the past for nucleofected human B cells, the introduction of pDNA *per se* may also induce rapid apoptosis, which was independent of the type (GFP vs. CD79b) and the presence of the transgene sequence but dependent on the DNA concentration, size, and circularity [43]. Similar observations were made for nucleofected murine splenic B cells [24]. Hence, besides the above-discussed cytotoxicity of GFP, the observed noxic effects could also be caused by the increased amounts of pDNA inside the cells, which would then constitute a more general problem in B cell transfection. 

Most probably, it is a mixture of the presence of large amounts of pDNA, high expression of the potentially toxic GFP, and most likely also the stripping of transcription factors (TFs) by the GFP expression cassette, which then are missing for the expression of other necessary cell proteins, which negatively influences the cell viability. Activated B cells, as used here for transfection, undergo significant and continuing transient changes in their metabolism, which makes them particularly sensitive towards inhibitory effects on transcription/translation [56].

### 2.5. Possible Effects of B Cell Polyclonality during Transfection

**Figure 5:**
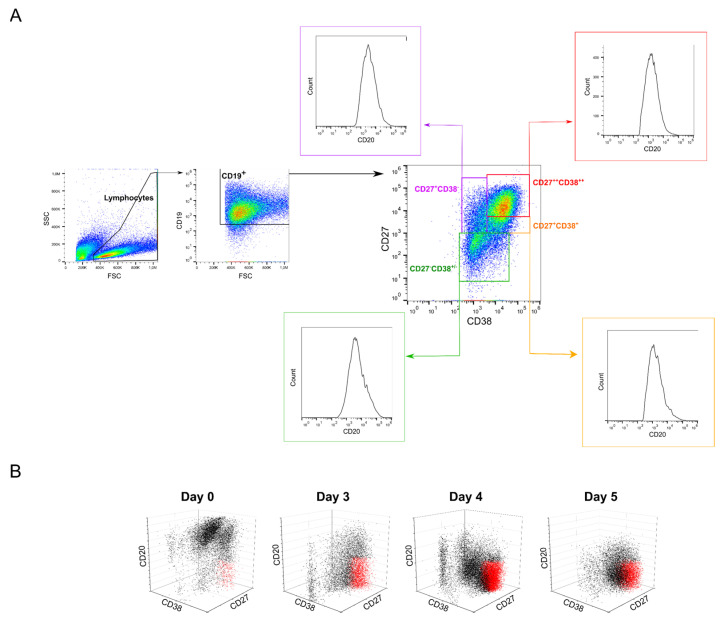
Evolution of the B cell population during the cultivation. (**A**) Gating strategy for the analysis of the B cell subsets. Naïve: CD19^+^CD20^+^CD27^−^CD38^−/+^ (green); Memory: CD19^+^CD20^+^CD27^+^CD38^−^ (purple); GC: CD19^+^CD20^−/+^CD27^+^CD38^+^ (orange); Plasma CD19^+^CD20^-^CD27^++^CD38^++^ (red) cells. (**B**) Representative 3-parameters analysis of B cell subpopulations on day of thawing (day 0) and after 3 to 5 days cultivation.

The tonsillar B cells used in this study consisted of several heterogeneously represented subpopulations distinguishable by their CD markers [57,58] (i.e., CD19, CD20, CD27, CD38) [39]. Moreover, a set of mitogenic factors was used to activate the B cells prior to transfection. The added rhCD40L, in particular, induced B cells proliferation, which was accompanied by additional differentiation into the B cell subtypes [59,60]. Post-transfection, the cells were again cultivated in activation medium, i.e., in the presence of rhCD40L. The expression of the transgene was, in our case, under the control of the CMV promoter. Therefore, the transcription was mainly regulated by the nuclear factor κB (NFκB) transcription factors [61,62]. CD40L is known to prompt an upregulation of the NFκB expression [63,64], which, as recently shown by Huse et al., differs in the B cell subsets and is tendentially lower in the GC cells compared to naïve and memory cells [65]. NFkB is also implicated in the regulation of the plasma cells [66]. Hence, the distribution of the B cells subsets on the day of transfection could differ, potentially influencing the cells’ sensitivity towards the transfection agent, but also in terms of the ensuing TE (transgene expression) under otherwise similar conditions. Each subpopulation may react differently in regard to polyplex uptake and transgene expression, leading in the end to major differences in the overall transfection outcome—An issue that had been overlooked in previous studies of primary B cells transfection, where experiments were performed with pools of B cells containing heterogeneous and variable cell populations. The typical evolution of the B cell subclasses during 0 to 5 days of cultivation in activation medium is presented in Figure 5 and Table 4. 

As evidenced by the flow cytometry analysis (Figure 5), the distribution of the B cells subpopulations changed markedly between days 3 and 5 of cultivation post-thawing, in particular in regard to the plasma cells (colored in red in Figure 5B), whose fraction increased until day 5 of cultivation. Moreover, whereas naïve and memory cell populations decreased during the cultivation/expansion in growth medium, the fraction of cells with GC signatures decreased during the first 3 days of cultivation and then increased again. Most prominently, the plasma cells population rose to more than 60% during that time (Table 4).

At present, we can only hypothesize that the distribution of B cell subsets is related to the observed variations in the transfection outcome, but we propose that discrete subsets respond differently to the transfection agent compared to others. In particular, the plasma cell blast phenotype (i.e., large cells in FSC/SSC) might play an important role by exhibiting a larger membrane area available for polyplexes interaction. As recently reported, a dilution of the electro-transfection buffer with water improved (≥2-fold) the transfection rate in a variety of B cells (i.e., lymphoblastoid, B-cell lines, and PBMCs), albeit with a concomitant decrease in cell viability [23]. Even though not discussed in this publication, it is tempting to assume that a gradual reduction in the salt concentration creates a hypotonic environment, which in turn might lead to swelling of the cells, accompanied by an increase in the cell membrane surface area, due to the alteration in osmotic pressure. Such an influence of the cellular membrane area on the transfection efficiency during electroporation has also been postulated for CHO cells [67]. Finally, it may be of significance in this context that the plasma cell fraction reached up to 50% during expansion, while most publications discussing B cells transfection report maximum TEs hardly exceeding 50% [22,24,43,49]. 

## 3. Conclusions

Despite the enormous progress in research and development of non-viral cell transfection methods, the delivery of nucleic acids into primary cells, particularly into immune cells, is still challenging. Here, we presented, for the first time, a nanostructure-based method, using star-shaped cationic DMAEMA-polymers for transfection, which is particularly promising for transfection of human primary B cells. Intensifying the contact between the polyplexes and the cells, fine-tuning polymer and plasmid quantities, as well as the cultivation time pre- and post-transfection, we were able to transfect human tonsillar B cells with 40% efficiency at reasonable cell viability (ca. 70%). Our results represent a significant improvement when compared to previously reported non-viral chemical transfection protocols. Moreover, when compared to Nucleofection, i.e., the current method of choice of non-viral gene delivery into B cells, the nano-stars-based method requires 500-fold fewer cells and about 370-fold less pDNA to reach comparable transfection efficiencies. Hence, this would allow performing more experiments with the cells from one biopsy and limit the need for large amounts of high-quality pDNA. Most importantly, our data suggest that the complexity of the B cell subpopulations (i.e., variation of the subsets’ distribution) pre- and post-transfection may be a critical parameter to consider. In the future, the role of the B cell subsets pre- and post-transfection needs to be studied in more detail to better understand their reactivity during the transfection process and hence improve the overall transfectability and viability of primary B cells under these circumstances. 

## 4. Materials and Methods

### 4.1. Materials

If not otherwise indicated, we used Greiner Bio-One (Frickenhausen, Germany) as the supplier for cell culture materials and Sigma-Aldrich (Taufkirchen, Germany) for chemicals. Linear PEI (l-PEI, 25 kDa) was from Polysciences (Polysciences Europe GmbH, Eppenheim, Germany). The nano-structure (“nano-star”) used as transfection agent was kindly provided by C.V. Synatschke (current affiliation: Max Planck Institute for Polymer Research, Mainz, Germany). This transfection agent is not commercially available but can be synthesized following a published protocol [37]. Fetal calf serum (FCS) was from Biochrom (Biochrom AG, Berlin, Germany). Dulbecco’s phosphate-buffered saline (DPBS) without Ca^2+^ and Mg^2+^ was from Lonza (Visp, Switzerland). Hanks balanced salt solution (HBSS) without Ca^2+^ and Mg^2+^ was from Biochrom (Biochrom AG, Berlin, Germany). HBG buffer (20mM Hepes, 5 wt % glucose, pH 5.5) was prepared in-house and sterilized by filtration (Chromafil^®^, CA-20/25(S), 0.2 µm; VWR, Ismaning, Germany). Cell culture media Roswell Park Memorial Institute 1640 (RPMI1640) and Iscove’s Modified Dulbecco’s Medium (IMDM) were from Lonza (Visp, Switzerland) and Sigma-Aldrich, respectively. Opti-MEM culture medium supplemented with GlutaMAX was from ThermoFisher Scientific (Dreieich, Germany). Erylysis buffer (0.155 M NH_4_Cl, 0.01 M KHCO_3_, 0.01 mM EDTA) was prepared in-house as 10× concentrated solution and sterilized by filtration (Chromafil ^®^, CA-20/25(S), 0.2 µm; VWR, Ismaning, Germany). For pre-equilibration, media were incubated for 1 h in a standard mammalian cell culture incubator (37 °C, 5% CO_2_, 95% humidity). For induction of the B cell proliferation the following medium was used (referred to as “growth medium”: 88% IMDM medium, 10% human AB serum, Cyclosporin A (CsA, 1 µg mL^−1^) all from Sigma-Aldrich (Taufkirchen, Germany), 1% Ultraglutamine (200 mM, Lonza, Visp, Switzerland), ITS-G (100×, ThermoFisher, Dreieich, Germany), Interleukin-4 (rhIL-4, 10 ng mL^−1^), Interleukin-21 (rhIL-21, 20 ng mL^−1^), B-cell activating factor (rhBAFF, 4 ng mL^−1^), rhCD40L (400 ng mL^−1^) all from Miltenyi Biotec (Gladbach, Germany). Tonsillar tissue as the source for the B cells was obtained during routine tonsillectomy (complete removal of the tonsillar tissue) (Gemeinschaftspraxis Gollner, Kulmbach, Germany). Written consent for the intended utilization was obtained, after verbal and written information on research goals, as approved by the ethical review committee from the University of Bayreuth, Germany (written approval #O 1305/1-GB, 2018).

### 4.2. Methods

#### 4.2.1. Cell Culture

B cells were isolated as previously described [39]. Briefly, after removal by surgery, tonsillar tissue was immediately transferred into an ice-cold buffer (HBSS containing 100 U mL^−1^ penicillin, 100 µg mL^−1^ streptomycin, 2.5 µg mL^−1^ amphotericin B, 2mM Ethylenediaminetetraacetic acid (EDTA), and 0.5% (*w/v*) bovine serum albumin (BSA)) and placed on ice for the transport. Upon arrival in the lab, the tonsillar tissue was immediately placed in RPMI1640 culture medium and cut into small pieces. The pieces were transferred into a 70 µm cell strainer (Greiner Bio-One, Frickenhausen, Germany) placed on a 50 mL centrifuge tube, and the material was pushed through the mesh with the help of a syringe piston. The remaining erythrocytes were lysed by incubation in Erylysis buffer (1×) for 5 min. Cell debris and any remaining red cells were removed by density gradient centrifugation (Ficoll LSM 1077; PAA Laboratories GmbH, Pasching, Austria) according to the supplier’s instructions. Mononuclear cells were collected and resuspended in HBSS containing 10% (*v/v*) heat-inactivated FCS (“HBSS-FCS”). A maximum of 4 × 10^8^ cells in 4 mL HBSS-FCS were applied to a sterilized 20 mL syringe column (B. Braun, Melsungen, Germany) packed with 1 g sterile nylon wool (Polysciences Inc., Hirschberg an der Bergstrasse, Germany) and incubated upright for 1 h in the cell culture incubator. Afterward, the non-bound cells (mainly T cells) were eluted by gently rinsing the wool twice with one column volume of HBSS-FCS. Thereafter, the B cells were collected by filling the column with fresh HBSS-FCS, followed by mechanical agitation to detach the cells. Subsequently, the wool was squeezed by pushing down the syringe piston to flush out the B cells. This step was repeated twice. B cells were recovered by centrifugation (300× *g*, 5 min) and resuspended in cryo-medium (90% FCS-10% DMSO) prior to cryopreservation. 

For the experiments, cells were thawed, and 1 mL of the obtained B cell suspension was washed with 9 mL DPBS. The cells were recovered by centrifugation (400× *g*, 10 min), the supernatant was discarded, and the cell pellet was resuspended in growth medium. The cells were then seeded at a cell density of 10^6^ cells mL^−1^ onto tissue culture plates (10 cm Petri dish) for expansion. Before transfection, the B cells were incubated at 37 °C (95% humidity, 5% CO_2_) in the growth medium without medium change for up to 6 days to induce proliferation. 

#### 4.2.2. Plasmid

Plasmid pEGFP-N1 (4.7 kb) used for polyplex formation was from Clontech Laboratories, Inc. (Mountain View, CA, USA). The plasmid encodes for an enhanced Green Fluorescent Protein (referred to as GFP) and was amplified in *Escherichia coli* using standard laboratory techniques (LB medium supplemented with 30 µg mL^−1^ kanamycin). The EndoFree Plasmid Kit (Giga Prep/Maxi Prep) from QIAGEN (Hilden, Germany) was used for plasmid preparation (quality control: >80% supercoiled topology (agarose gel) and A_260_/A_280_ ≥ 1.8). Purified plasmids were solubilized in sterile ultrapure PCR-water (Sigma-Aldrich, Taufkirchen, Germany).

#### 4.2.3. Transfection

*Polycationic Transfection Agents*

Besides the l-PEI from Polysciences, a well-defined star-shaped polymer (referred to as nano-star) synthesized in-house via atom-transfer radical polymerization (ATRP) of DMAEMA, was used as polycationic transfection agent. Synthesis and characterization of the nano-star have been described previously [31,37]. An average nano-star consists of an inorganic core decorated with 24 polycationic PDMAEMA arms, each with an average length of 230 monomeric units, see structure below. The number average molecular weight, Mn, of the construct was 755 kDa, and the polydispersity (Mw/Mn) was <1.21.

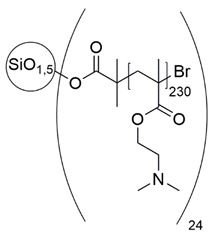



Polymer stock solutions were prepared in sterile ultrapure PCR-water (Sigma-Aldrich, Taufkirchen, Germany) as 1.25 mg mL^−1^ (l-PEI) and 1.82 mg mL^−1^ (nano-star) and diluted for use as indicated. LD_50_ values were 12.1 μg mL^−1^ for l-PEI and 500 μg mL^−1^ for nano-star as previously determined by a standard MTT assay [68] using L929 cells [32,37].

*N/P-Ratio Calculation*

N/P-ratios were calculated according to: 

N/P = $\frac{µL\;polycation\;stock\;solution\times N}{\text{µ}g\;pDNA\times 3}$

With N concentration (mM) of nitrogen residues in the transfection agent. Note: 1 µg of DNA contains 3 nmoles of anionic phosphate.

*Transfection Protocols*

Prior to transfection, cells were cultivated in growth medium to induce B cells proliferation. Usually, 3 to 6 days were necessary to produce a sufficient number of cells for transfection. On the day of transfection, the cells were collected by centrifugation (400× *g*, 10 min) and washed twice with 10 mL DPBS. After resuspension in Opti-MEM, cell count and viability were determined with a LUNA-FL™ Dual Fluorescence Cell Counter (Logos Biosystems, Gyeonggi-do, South Korea). Thereafter, the cells were transfected with l-PEI or nano-stars according to the procedures described below.

*6-Well plate transfection protocol*

1 mL of the cell suspension (2 × 10^5^ cells mL^−1^ in OPTI-MEM) was transferred into a well of a 6-well plate and incubated (37 °C, 95% humidity, 5 % CO_2_) while the polyplexes were being prepared. For polyplex preparation, the first 3 µg of pDNA was mixed with HBG-buffer unless otherwise mentioned. The mixture was vortexed for approximately 1 sec before the amount of polymer needed for the intended N/P ratio was added in a single drop. Immediately after, the mixture (200 µL) was vortexed for exactly 10 sec at 2200 rpm and incubated at room temperature. After 20 min of incubation, 800 µL of Opti-MEM was added per 200 µL of polyplex solution followed by incubation for 10 additional min at room temperature.

1 mL of the polyplex solution was added to the cell suspension in the 6-well plate (total volume per well then: 2 mL) and incubated (37 °C, 95% humidity, 5% CO_2_) for 240 min. Afterward, the cell/polyplex mixture was transferred to a micro-tube, and the cells were separated from the supernatant by centrifugation (400× *g*, 10 min). The supernatant was discarded, the cell pellet was suspended in 500 µL of growth medium by gently pipetting up and down and transferred to the well of a 24-well plate. The tube was then rinsed with 500 µL growth medium, which was added to the corresponding well (Total cultivation volume: 1 mL). The plate was placed in the cell culture incubator for up to 48 h.

*Tube transfection protocol*

1 mL of the cell suspension (2 × 10^5^ cells mL^−1^ in OPTI-MEM) was transferred into a micro-tube and stored on ice while the polyplexes were being prepared. In this protocol, the N/P ratio was adjusted by varying the amount of pDNA while keeping the polymer amount constant. For polyplex preparation, first, HBG-buffer was added, followed by a suitable amount of pDNA needed for the intended N/P ratio. The mixture was vortexed for approximately 1 sec before the required amount of transfection agent was added in a single drop. Immediately after, the polyplex solution (50 µL) was vortexed for exactly 10 sec at 2200 rpm. The mixture was incubated at room temperature for 20 min, followed by the addition of 450 µL of Opti-MEM per 50 µL of polyplex solution. This was followed by another 10 min incubation at room temperature.

The cells stored on ice were recovered by centrifuged (400× *g*, 10 min), and the supernatant was discarded. The cell pellet was mechanically dislocated prior to adding the polyplex/Opti-MEM mixture. Cells and polyplexes were gently mixed before placing the tube upright in the cell culture incubator (37 °C, 95% humidity, 5% CO_2_) for up to 90 min. After the indicated time span, the cells were recovered by centrifugation (400× *g*, 10 min), the supernatant was discarded, and the cell pellet was resuspended in 500 µL of growth medium. After mixing in by gently pipetting up and down, the cell suspension was transferred into the well of a 24-well plate. The tube was then rinsed with 500 µL growth medium, which was added to the corresponding well (total cultivation volume: 1 mL). The plate was placed in the cell culture incubator for up to 48 h.

To investigate the influence of the transfection procedure per se, aliquots of the cells were always put through a mock transfection (referred to as “Mock”), i.e., were solely incubated with the complexation buffer.

#### 4.2.4. Analytics

*Determination of cell number and viability*

A LUNA-FL™ Dual Fluorescence Cell Counter (Logos Biosystems, Gyeonggi-do, South Korea) was used to determine the number and viability of the cells. For this purpose, the cells were stained with an Acridine Orange (AO, staining all cells)/Propidium Iodide (PI, staining dead cells) solution (Logos Biosystems, Gyeonggi-do, South Korea) according to the supplier´s instructions.

*Determination of the transfection efficiency (GFP fluorescence) and survival rate*

The transfection efficiency (TE) was assessed by flow cytometry (Cytomics FC500; dual laser (488nm, 635 nm); Beckman Coulter, Krefeld, Germany). Forward scatter (FSC), side scatter (SSC), green fluorescence (FL1, 525 nm, GFP), and red fluorescence (FL3, 620 nm, PI) were recorded, the FL1 and FL3 signals on a logarithmic scale. Twenty-four hours to 48 h post-transfection, cells were recovered by centrifugation (400× *g*, 5 min) and resuspended in DPBS containing 1 µg mL^−1^ propidium iodide (PI) to counterstain the dead cells (for the viability measurements). Negative controls, i.e., non-transfected cells, were used to set the measurement parameters. Data were collected from at least 50,000 events. Lymphocytes can be distinguished based on their forward (FSC) and sideward (SSC) light scatter properties, which reflects their size and their relatively agranular cytoplasm [69]. Cells were, therefore, initially evaluated by scatter properties (FSC/SSC) in order to select the lymphocytes population (Gate: “Lymphocytes”). The relative GFP fluorescence of the gated cells was quantified, allowing a statistical quantification of the percentage of transfected cells (“transfection efficiency”, TE) in the lymphocyte population. Concomitantly, this cell population was analyzed for red fluorescence intensity (PI) to determine cell viability. Histogram plots of the respective fluorescence intensities (log scale) were used to estimate the expression level of GFP (Median, MFI). We defined GFP-expressing cells as cells having a fluorescence higher than the fluorescence of the non-transfected cells (i.e., autofluorescence of the cells). The gating strategy for the analysis of the transfected lymphocytes is presented in Figure 1. Flow cytometry data were evaluated using FlowJo software v 10.7.1 (Tree Star, Stanford University, Stanford, CA, USA, 2016).

*B cell subtype analysis (phenotyping)*

For phenotyping, cell surface markers (CD19, CD20, CD27, and CD38) characteristic for certain B cell subsets were assessed by flow cytometry after staining the cells with CD-specific antibodies (anti-CD19-APC, #302212; anti-CD20-PE, #302306; anti-CD27-PE-Cy7, #356412; anti-CD38-FITC, #356610; all from BioLegend, San Diego, CA, USA) according to the manufacturer’s instructions. Briefly, 5 × 10^5^ cells were washed twice in 1 mL DPBS (400× *g*, 5 min) and resuspended in 100 µL DPBS before incubation with the antibodies for 30 min on ice. Subsequently, cells were washed twice with 1 mL DPBS (400× *g*, 5 min) and resuspended in 500 µL DPBS prior to analysis. Flow cytometric measurements were set to 80,000 events in total. Control cells (i.e., treated as the immune-stained cells but without antibody addition) were used to set the measurement parameters. Forward scatter (FCS), side scatter (SSC), and fluorescence intensity (FITC emission 525 nm, PE emission 575 nm, APC emission 655 nm, PE-Cy7 emission 750 nm) were recorded. For analysis, the lymphocyte population was gated according to its scattering properties as indicated above (Gate: “Lymphocyte”). The cells in the “Lymphocyte” gate were further analyzed for the presence of the surface markers. Within the “Lymphocyte” population, a gate “B cells” was defined as containing the CD19 positive cells, i.e., all cells carrying the pan-B antigen (CD19^+^). The gating strategy for the analysis of the B cell subsets is presented in Figure 5A. Flow cytometry data were evaluated using the FlowJo software as indicated above.

#### 4.2.5. Statistical Analysis

Group data are reported as mean ± standard deviation. If not otherwise stated, n represents the number of independent experiments. OriginPro software (version 2021, OriginLab, Northampton, MA, USA) was used for One-way ANOVA with Holm-Sidak multiple comparison tests to determine whether data groups differed significantly from each other. Statistical significance was defined as *p* < 0.05.

## Figures and Tables

**Table 1:** Transfection of human primary B cells with PDMAEMA-nano-stars and l-PEI using the standard transfection method.

	TE ^1^ (%)		MFI ^2^ (a.u.)		Viability (%)	
N/P Ratio	Nano-Stars 24 h/48 h	l-PEI 24 h/48 h	Nano-Stars 24 h/48 h	l-PEI 24 h/48 h	Nano-Stars 24 h/48 h	l-PEI 24 h/48 h
3	4.9/2.4	3.7 /0.1	750/1085	495/715	66.3/48.8	90.9/95.9
5	9.3/6.5	1.8 /0.2	1289/2393	483/732	62.1/58.3	95.8/88.1
7.5	7.5/7.2	5.0 /0.3	1719/2099	500/732	60.9/54.3	77.7/83.9
12.5	24.3/8.1	1.9 /0.2	947/1311	496/784	46.6/44.9	80.5/90.5
15	15.1/6.2	2.7 /0.3	997/1096	503/778	52.9/31.2	75.1/93.8
20	20.5/4.1	4.1 /0.1	944/1103	500/730	55.5/42.3	80.7/87.3

^1^: TE, transfection efficiency. ^2^: MFI, median fluorescence intensity. Transfection in 6-well plates at day 4 post-thawing, pDNA: 3 µg, N/P ratio adjusted by varying the amount of polycation. Cell number for transfection: 2 × 10^5^ cells. Contact time: 4 h. Transfection volume: 2.0 mL, *n* = 1. Cell viability on the day of transfection: >80%. a.u.: arbitrary units.

**Table 2:** Influence of the batch variation on transfection efficiency and viability.

Cryovial Nb.	Viability Pre-Transfection (%)	Transfection Efficiency (%)	Viability Post-Transfection (%)
I ^1^		22	33
	79.9	28.6	35.6
		28	35.4
II	90.4	41.3	54.1
III	84.4	16	46.2
IV	86.9	31.8	52.9
V	82.6	40.8	63.6
Mean ± SD (%)	83.4 ± 4.1	29.8 ± 9.2	45.8 ± 11.6

Transfection in tubes. Cell number during transfection: 2 × 10^5^ cells (day 4 post-thawing). Polymer density: 15 µg per 10^6^ cells, polymer concentration: 6 µg mL^−1^, pDNA: 0.6 µg per tube. Contact time: 30 min. N/P: 10, transfection volume: 0.5 mL. TE and viability were measured 48 h post-transfection. *n* = 1. ^1^: Technical replicates, mean_TE_: 26.2 ± 3.6 % and mean_viability_: 34.7 ± 1.4 %.

**Table 3:** Transfection outcomes as a function of the pre- and post-transfection cultivation time.

	Cultivation Time				
	Pre-Transfection (Days)	Post-Transfection (Hours)	TE (%)	MFI ^2^ (a.u.)	Viability (%)
Tf_d3_	3	24	39.2 ± 0.9	34,537 ± 1532	61.8 ± 0.3
			(*n* = 3)		
		48	28.2 ± 6.1 *	32,220 ± 2695	53.0 ± 1.6 *
			(*n* = 3)		
Tf_d4_^1^	4	24	37.4 ± 1.8	41,564 ± 11,966	69.3 ± 1.7 ^#^
			(*n* = 2)		
		48	32.5 ± 3.9	35,204 ± 9644	54.2 ± 2.4 *
			(*n* = 4)		
Tf_d5_	5	24	n.a	n.a	n.a
		48	21.2 ± 5.8 ^§^	10,940 ± 759	38.3 ± 9.8 ^#^
			(*n* = 3)		

^1^: For analytical sake, some of the transfection data included in the calculation were identical to the one presented below in § 2.4. ^2^: MFI, median fluorescence intensity. 2 × 10^5^ cells per samples; N/P 10; 15 µg polymer per 10^6^ cells; 6 µg polymer per mL. Contact time: 30 min. Transfection in tubes, transfection volume: 0.5 mL. Cell viability on the day of transfection: >80%. Data represent mean values ± SD. n.a.: not available. Statistical significance between “day of cultivation pre-transfection” groups is indicated as ^#^ (*p* < 0.05). Statistical significance between Tfd5 and Tfd3 or Tfd4 is indicated as ^§^ (*p* < 0.05). Statistical significance between “24 h” and “48 h” groups is indicated as * (*p* < 0.05). See Figure S2 for representative histograms of GFP expression levels 48 h post-transfection. a.u.: arbitrary units.

**Table 4:** Distribution of the B cell ^1^ subclasses (%) as a function of the cultivation time post-thawing.

		Total Cultivation Time (Days)			
Subclasses	Classification ^2^	0	3	4	5
CD20^+^CD27^−^CD38^−/+^	Naive	21.0 ± 0.1	7.7	7.9 ± 3.7	2.7 ± 0.7
CD20^+^CD27^+^CD38^−^	Memory	24.6 ± 5.8	7.4	4.1 ± 2.6	0.6 ± 0.04
CD20^−/+^CD27^+^CD38^+^	GC ^3^	12.8 ± 1.0	3.9	6.1 ± 2.5	7.0 ± 4.4
CD20^−^CD27^++^CD38^++^	Plasma	2.8 ± 0.6	43.1	55.2 ± 6.1	69.0 ± 10.6

^1^: Gate “Lymphocytes” and subgate CD19^+^-cells for flow cytometry analysis. ^2^ Classification according to Jackson et al. [57,58]. ^3^ GC: germinal center. The data represent the subclasses distribution within the CD19^+^-cells in the “lymphocyte” gate as mean values ± SD, *n* ≥ 2 (for day 3, *n* = 1). Gating strategy and population distribution in a 3-parameters plot are presented in Figure 5.

## Data Availability

All data are available from the corresponding author upon reasonable request.

## Supplementary Materials

The following are available online at https://www.mdpi.com/article/10.3390/ijms22158239/s1, Figure S1: Growth curve of human primary B cells during expansion in growth medium, Figure S2: Representative histograms of GFP expression 48 h post-transfection, Figure S3: Correlation between the level of GFP expression and the level of propidium iodide (PI) incorporation, Table S1: Polymer densities and polymer concentrations used for the transfection under standard conditions (6-well plate protocol), Table S2: Transfection of human primary B cells with l-PEI using the tube transfection protocol.
