# Supplementary material for: Generation of Recombinant Primary Human B Lymphocytes Using Non-Viral Vectors
Source: Int J Mol Sci. 2021 Jul 30;22(15):8239. doi: 10.3390/ijms22158239 (PMC8347318; doi:10.3390/ijms22158239)
Supplement: Supplementary file 1 [file ijms-22-08239-s001.zip › ijms-1320112-supplementary.pdf]

Supplementary information

Generation of recombinant primary human B lymphocytes using non-viral vectors

Daniel Keim [1], Katrin Gollner[2], Ulrich Gollner[2], Valérie Jérôme [1] and Ruth Freitag [1,*]

[1] Process Biotechnology, University of Bayreuth, Germany; daniel.keim@uni-bayreuth.de, valerie.jerome@uni-bayreuth.de
[2] Praxis am Schießgraben, Schießgraben 21, 95326 Kulmbach, Germany; gollner@hno-operationen.de
* Correspondence: ruth.freitag@uni-bayreuth.de

Supplementary Figures

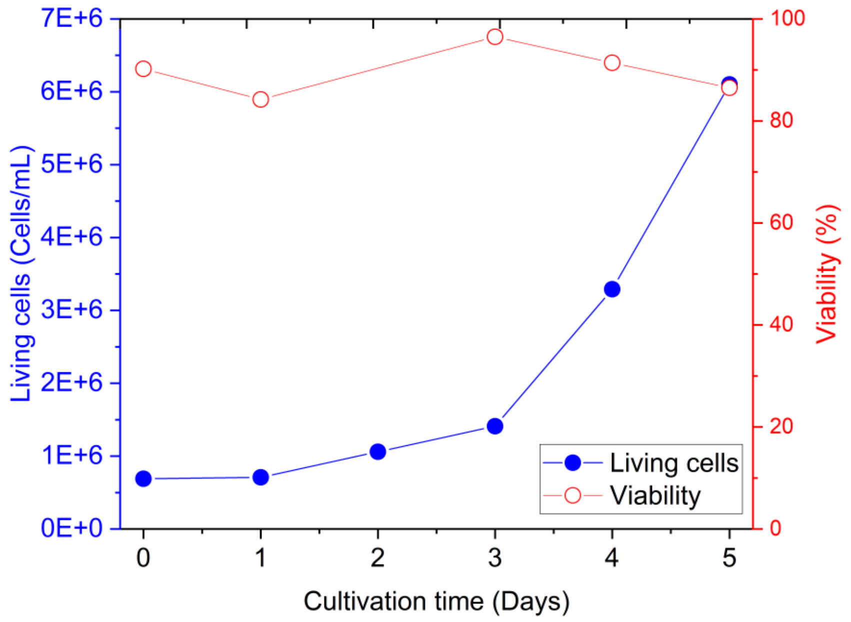

Figure S1: Growth curve of human primary B cells during expansion phase in growth medium Representative growth curve. The cells were cultivated for up to five days in tissue culture plates (10 cm Petri dish) for expansion. Growth medium: IMDM medium supplemented with 10 % human AB serum, 1 µg mL^{-1} cyclosporin A, 2 mM ultraglutamine, 1X ITS-G, 10 ng mL^{-1} interleukin-4, 20 ng mL^{-1} interleukin-21, 4 ng mL^{-1} BAFF, 400 ng mL^{-1} rhCD40L.

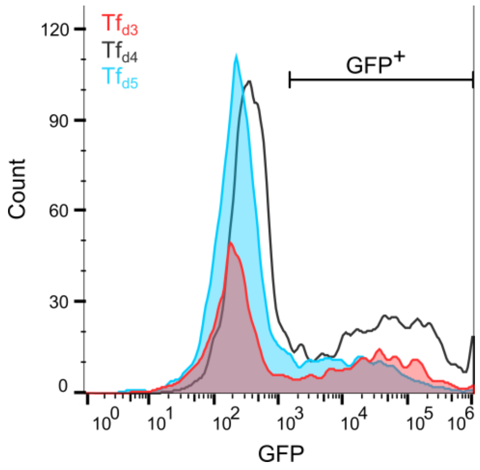

Figure S2: Representative histograms of GFP expression 48 h post-transfection The cells were transfected after three to fives days of cultivation post-thawing.

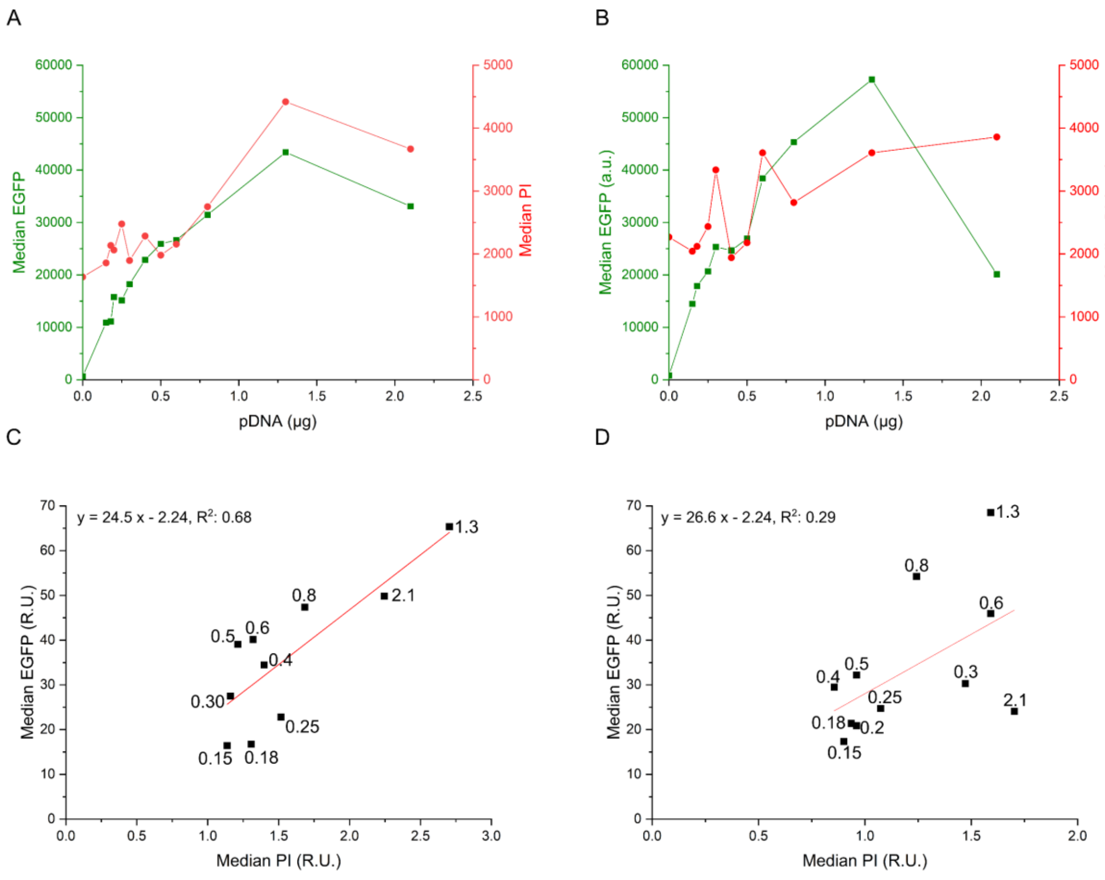

Figure S3: Correlation between GFP expression levels and levels of propidium iodide (PI) incorporation

A, B: GFP expression level (green square) and level of PI incorporation (red circle). C, D: Relative median values (normalized with mock-transfected cells values) corresponding to the data presented in Figure 4. Incubation post-transfection: 24 h (A, C), 48 h (B, D). Amount of pDNA (µg per tube, 2 x 10^5 cells) used during transfection is given next to the corresponding data point.

Supplementary Tables

Table S1: Polymer densities and polymer concentrations used for the transfection under standard procedure (6-well plate protocol).

N/P ratio	Polymer density (µg per 10^6 cells)		Polymer concentration (µg mL^{-1})	
	Nano-star	l-PEI	Nano-star	l-PEI
3	21.6	5.8	2.2	0.6
5	35.9	9.7	3.6	1.0
7.5	53.9	14.6	5.4	1.5
12.5	89.8	24.3	9.0	2.4
15	107.8	29.1	10.8	2.9
20	143.7	38.8	14.4	3.9

Transfection in 6-well plates, pDNA: 3 µg per tube corresponding to 1.5 µg mL^{-1} and 15 µg per 10^6 cells, N/P ratio adjusted by varying the amount of added polymer. Cells number during transfection: 2 x 10^5 cells. Transfection volume: 2.0 mL.

Table S2: Transfection of human primary B cells with l-PEI using the tube transfection protocol.

N/P ratio	Amount of pDNA/tube	TE[1] (%)	Viability (%)
"Mock"	7.7	0	96.9
3	4.6	0	91.9
5	3.1	0	85.9
7.5	2.3	0.1	85.9
10	1.9	0.1	82.1
12.5	1.6	0.1	93.2
15	1.2	0.2	84.5
20	0.9	0.3	78.1
35	0.7	0.2	85
40	0.6	0.2	73

[1]: TE, transfection efficiency. Polymer density: 15 µg per 10^6 cells, polymer concentration: 6 µg mL^{-1}, N/P ratio adjusted by varying the amount of pDNA. Transfection day 4 post-thawing. Cell number during transfection: 2 x 10^5 cells. Contact time: 30 min. Transfection volume: 0.5 mL. n = 1. Cell viability on the day of transfection: > 80%. TE and viability measured 48h post-transfection. "Mock": cells solely incubated with the complexation buffer.
